# Acceptability and Effectiveness of Text Message Reminders to Improve Patient Attendance During the Sociopolitical Crisis in Haiti: Telephone-Based Survey

**DOI:** 10.2196/77010

**Published:** 2026-02-23

**Authors:** Marcmy Presume, Charles Patrick Almazor, Jean Rony Quetant, Mathias Altmann

**Affiliations:** 1 Bordeaux Population Health Research Centre, Research Institute for Sustainable Development (IRD), EMR 271, National Institute for Health and Medical Research (INSERM), UMR 1219, University of Bordeaux, 146 rue Léo-Saignat, Bordeaux, 33076, France, 33 0557571580; 2 Hopital Sage Citymed, Port-au-Prince, Haiti; 3 Jerusalem Clinique Medicale, Port-au-Prince, Haiti

**Keywords:** patient appointment, reminder systems, text messaging, sociopolitical crisis, haiti

## Abstract

**Background:**

The sociopolitical crisis in Haiti affects health care center attendance, creating significant challenges in ensuring patient compliance with medical appointments.

**Objective:**

This study aimed to assess the effectiveness of text message reminders on patient attendance in the context of the sociopolitical crisis of Haiti, examining the influence of patient perceptions, behaviors, and socioeconomic factors.

**Methods:**

We conducted a cross-sectional study using a telephone survey of 386 randomly selected patients who had an appointment during the third quarter of 2024 at 2 health care centers in the Port-au-Prince metropolitan area. We collected appointment and socioeconomic data, as well as perceptions and behaviors toward text message reminders. We described patients’ perceptions and behaviors toward text message reminders, as well as appointment attendance and patient characteristics. We performed bivariate and multivariate logistic regression models to assess whether receiving text message reminders and socioeconomic factors influenced overall appointment attendance.

**Results:**

Among 386 patients, 259 attended their appointments on either the appointment day or at a later date for an overall attendance rate of 67.1 % ( 95% CI 62.4%-71.8%). Attendance rates were higher among the 147 patients who confirmed receiving a reminder (77.6%) compared to the 239 who did not (60.7%). SMS text messaging reading behavior varied among patients. Overall, 219/386 (56.7%) patients always, 66/386 (17%) often, 75/386 (19%) sometimes, 21/386 (5%) rarely, and 5/386 (1%) never read their SMS text messaging. All patients liked the initiative of sending reminders and found them helpful. In the multivariate analysis, patients who confirmed receiving a reminder were more likely to attend their appointment compared to those who did not (adjusted odds ratio [AOR] 2.0, 95% CI 1.18-3.39). A patient satisfaction rate of 8 or higher with their physicians was significantly associated with higher attendance rates, compared to 6 or lower, with AORs increasing with satisfaction. Travel time less than 30 minutes (AOR 2.31, 95% CI 1.03‐5.19) and 30-60 minutes (AOR 2.78, 95% CI 1.24‐6.21), and being with a chronic disease (AOR 0.42, 95% CI 0.23-0.79) were also associated with appointment attendance*.*

**Conclusions:**

Our study highlights the potential of text message reminders to improve appointment attendance in Haiti, despite the sociopolitical crisis. The overall acceptability and positive perceptions of SMS text messaging reminders suggest that they can be a valuable tool in health care settings, especially when adapted to the local context. We recommend that health care centers in Haiti consider integrating SMS text messaging reminder systems into routine patient management to enhance adherence and optimize care delivery.

## Introduction

Haiti is facing a profound sociopolitical crisis characterized by escalating gang violence, political instability, and widespread displacement. As of early 2025, it was reported that 85% of the Port-au-Prince metropolitan area was controlled by armed gangs, resulting in the displacement of approximately one in ten of the population [1]. The crisis has a significant impact on the health system. By early 2024, 60% of the health care centers across Haiti were unable to function, and up to 20% of health professionals had left the country [2,3]. Increasing insecurity and the high risk of kidnapping affect health care center attendance, creating significant challenges in ensuring patient adherence and compliance with scheduled medical appointments.

While structural barriers like transportation and roadblocks may cause missed appointments, these factors do not fully explain the high rates of nonattendance observed. In many cases, patients simply fail to prioritize their health appointments due to competing daily demands, forgetfulness, or a lack of perceived urgency [4,5]. Improving attendance therefore requires interventions that address not only logistical obstacles but also behavioral and motivational factors by targeting the proportion of missed visits not caused by uncontrollable external barriers.

Text message reminders have proven to be a useful tool for addressing the challenges of medical appointment compliance in a variety of settings and contexts. Studies in low- and middle-income countries have shown that text message reminders significantly improve antenatal and postnatal care attendance rates, as well as childhood vaccination coverage [6,7]. Text message reminders were found to be effective in reducing nonattendance among patients requiring long-term follow-up for chronic illnesses [8]. Mobile phones are very popular in low- and middle-income countries; studies have shown that the majority of patients often read their text messages and are willing to receive health-related information [9-11].

Haiti has relatively wide mobile network coverage, with approximately 90% of the population residing in areas served by a mobile signal [12,13]. Given that adults constitute less than 60% of the total population, the country recorded a telephone subscription rate of 65 subscriptions per 100 inhabitants in 2022, according to the most recent data available from the World Bank [14,15]. Considering the popularity and accessibility of mobile phones among the population, text messaging may represent an effective tool to address low health care center attendance during periods of sociopolitical crisis.

In Haiti, a first study assessed the effectiveness of text message reminders to improve patient attendance [16]. Using administrative data, the authors accounted for patients showing up on the appointment day only but did not control for socioeconomic factors. Through this study, we aimed to go further, examining the influence of patient perceptions, behaviors, and socioeconomic factors to assess the effectiveness of text message reminders on patient attendance in the context of the sociopolitical crisis of Haiti in 2024.

## Methods

### Study Setting

Two health care centers implementing a patient reminder system decided to follow up on a primary study published in 2024 that assessed the effectiveness of the patient reminder system. They followed the recommendation of this study and decided to go further by conducting a telephone survey among patients who had an appointment during the third quarter of 2024 in order to assess the effectiveness of text message reminders on patient attendance, examining the influence of patient perceptions, behaviors, and socioeconomic factors. These 2 health care centers are located in lower Delmas, near the stronghold of one of the most feared gangs in the Port-au-Prince metropolitan area. One is a 37-bed facility that serves approximately 2000 patients per month and provides a package of services, including internal medicine, surgery, orthopedics, pediatrics, gynecology, emergency care, medical laboratory, pharmacy, and imaging. The other is an outpatient clinic that serves approximately 350 patients per month and provides a package of services, including internal medicine, surgery, orthopedics, pediatrics, gynecology, urology, ophthalmology, medical laboratory, and pharmacy. Patients initially arrive at health care centers as drop-in visitors, and physicians schedule follow-up appointments using the reminder system.

### Study Design and Sample Size Calculation

Between December 2024 and January 2025, we conducted a cross-sectional study using a telephone survey of a random sample of patients who had an appointment during the third quarter of 2024 at 2 health care centers in the Port-au-Prince metropolitan area to assess the effectiveness of text message reminders on patient attendance in the context of the sociopolitical crisis of Haiti, examining the influence of patient perceptions, behaviors, and socioeconomic factors. Text message reminders were sent via email or SMS text messaging to patients before their appointment day, using an automated reminder system. Email reminders incurred no cost to either the health care centers or the patients; however, not all patients had access to an email address. SMS text messaging reminders were free for patients but involved a cost to the health care centers. To facilitate the delivery of SMS text messaging reminders, system administrators were required to recharge the SMS text messaging account. All patients had a phone number, but not all received SMS text messaging reminders, as system administrators did not consistently recharge the SMS text messaging account in a timely manner [16]. The primary outcome was overall attendance, defined as attendance at either the scheduled appointment or a later date. We examined patient perceptions and behaviors regarding text message reminders and compared overall attendance rates between those who confirmed receipt of text message reminders prior to their appointment day and those who did not, controlling for socioeconomic factors.

Sample size was calculated using confidence interval methodology with a confidence level of 95% and a margin of error of 5%, as recommended for cross-sectional studies and surveys and commonly applied in epidemiological research [17,18]. Given the sociopolitical crisis and resulting population instability due to displacement, which made the nonresponse rate difficult to predict, we aimed for the largest feasible sample size and adopted the maximum-variability assumption (50%), representing the most conservative approach given the uncertainty in population parameters (Eq. 1) [19]. The required sample size was estimated to be 386, ensuring adequate precision to detect meaningful associations between attendance, receipt confirmation of reminder, and socioeconomic factors.


(1) $$n=\left(\frac{z^{2}\;X\;p\left(1-p\right)}{\epsilon^{2}}\right)$$

Where n is the sample size, *z* is the *z* score (1.96) associated with a 95% CI, *ϵ* is the margin of error (5%), and *p* is the overall population attendance rate (the conservative estimate of 50%).

### Data Collection

The survey was conducted via structured telephone interviews. Nurses from the respective health care centers were trained and served as interviewers to enhance the acceptability of the survey among patients. The survey was administered by the trained interviewers using a standardized questionnaire to minimize interviewer bias and ensure consistency in data collection. The questionnaire and survey tools were bilingual, with all components in both French and Haitian Creole. The eligibility criteria were having a medical appointment during the third quarter of 2024 (July-September) at the two selected health care centers in the Port-au-Prince metropolitan area. A randomly sorted list of 1682 eligible patients was used as the sampling frame. Patients were contacted by telephone and enrolled progressively, following the order of the randomly sorted list, until the target sample size was reached. This strategy follows the principle of simple random sampling, in which each eligible patient has an equal and independent chance of being selected. In addition, this approach offers the advantage of ensuring that the targeted sample size can be reached despite an unexpected nonresponse rate, as the list can simply be followed until the required number of participants is enrolled. This method provided flexibility in addressing the unpredictable nonresponse rate due to the sociopolitical crisis context. Informed consent was obtained from the participants or their parents. Kobo (Harvard Humanitarian Initiative) was used to facilitate data entry, allowing real-time data quality monitoring.

Data collected in the survey included attendance at the scheduled appointment, late attendance (voluntarily return at a later date after missing the original appointment), time to voluntarily return at a later date after missing the original appointment (in days), reasons for late or nonattendance, receipt confirmation of reminder (did the patient receive a reminder before their appointment day), means of reminder (SMS text messaging or email), SMS text messaging reading frequency, Patient’s perception toward reminders (liking text message reminder initiative, whether reminders were helpful or not, and means of reminder preference), travel time (number of minutes spent by the patient from their home to reach the health care center), modes of transportation used by the patient, financing medical expenses (whether the patient took in charge their medical expenses or did they receive financial support from someone else), perceptions of service costs (did the patient find service costs affordable, reasonable, or expensive), satisfaction with the physician (rated on a scale of 1-10, where 1 is very dissatisfied and 10 is very satisfied), occupation of respondents (occupation of adults and children’s parents), education level of respondents (education level of adults and children’s parents), and being with a chronic disease (defined as an illness that requires ongoing medical care, based on the patient’s self-report of whether they endure from a chronic condition).

We used appointment data from the reminder system as well. These data included means of reminder and sending status (whether a reminder was sent or not and via which means), day and time at which SMS text messaging reminders were sent, time to appointment (number of days between the scheduled date and the appointment date), patient age, patient gender, and having children (for adults and children’s parents).

### Statistical Analysis

We described patient perception and behavior toward SMS text messaging reminders, as well as appointment attendance and patient characteristics. We performed bivariate and multivariate regression analyses using logistic regression models to assess whether receiving text message reminders and socioeconomic factors impacted overall appointment attendance. A 2-way stepwise analysis based on the Akaike Information Criterion was performed to select the best model for the multivariate analysis.

We conducted a sensitivity analysis to explore nonresponse bias using appointment data from the reminder system. Nonresponse rates were compared across reminder groups, clinical specialties, gender, and age groups using the Pearson chi-square test ( Multimedia Appendix 1).

All statistical analyses were performed using Python (version 3.12.8; Python Software Foundation) with the following packages: statsmodels (for logistic regressions), SciPy (for Pearson chi-square test), matplotlib (for plots and graphs), seaborn (for plotting the heatmaps), pandas (for data manipulation and descriptive analysis), and numpy (for numerical operations and array handling). The Strengthening the Reporting of Observational Studies in Epidemiology (STROBE) checklist (Checklist 1) was used to report the findings [20].

### Ethical Considerations

Ethics approval for the study was granted by the Haiti Ministry of Health National Bioethics Committee (#2425‐8). Informed consent was obtained verbally from all participants or their parents. Participation was entirely voluntary, and participants were informed of their right to withdraw from the study at any time without any impact on their access to medical care or other services. Patient information was anonymized and deidentified before being handed over for analysis. No compensation was provided to participants. All methods were carried out in accordance with relevant guidelines and regulations.

## Results

### Overview

Between December 2024 and January 2025, 386 patients participated in the survey, resulting in a response rate of 63.0%. Of these patients, 259 attended their appointments on either the appointment day or at a later date for an overall attendance rate of 67.1% (95% CI 62.4%-71.8%).

### SMS Text Messaging Reading Behavior and Reminder Receipt Confirmation

**Figure 1.**
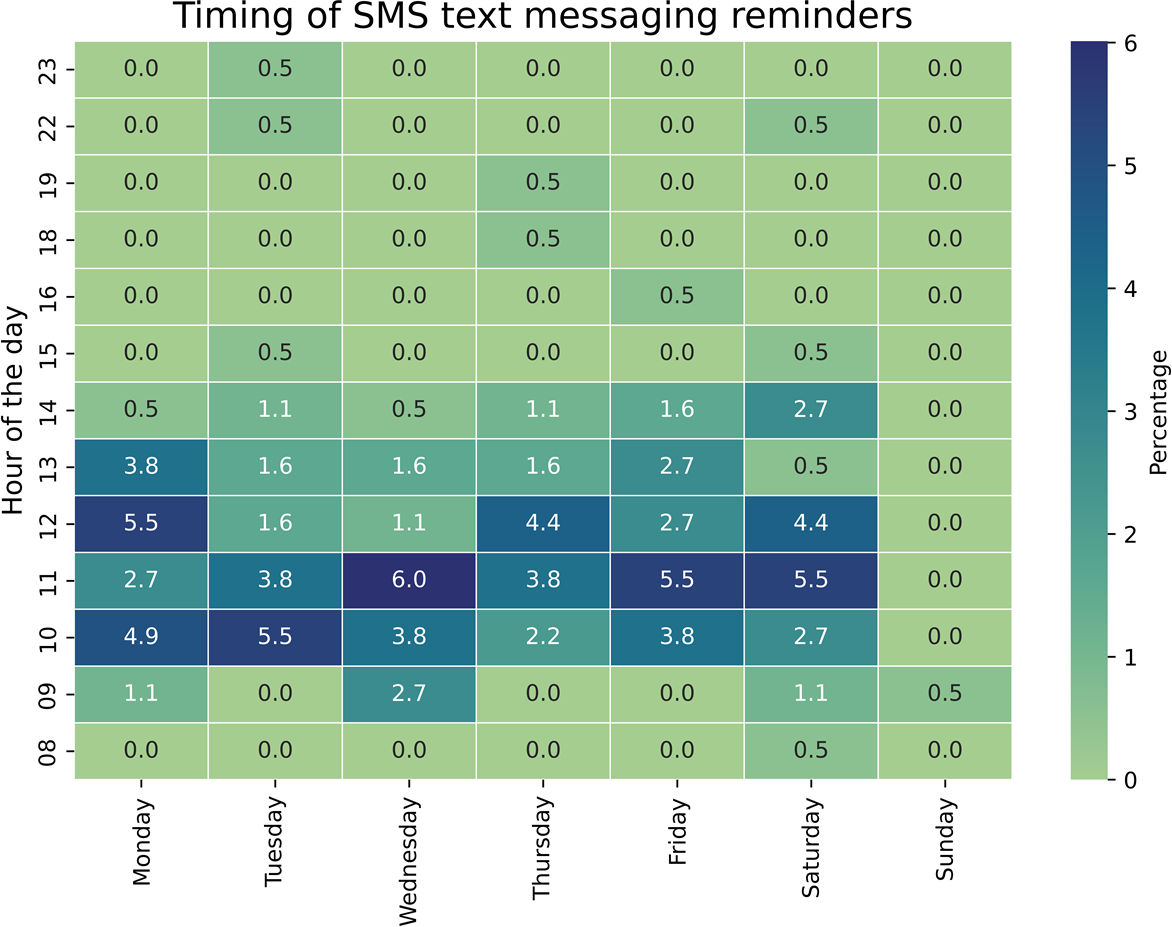
Percentage of SMS text messaging sent by hour of the day, Port-au-Prince metropolitan area, 2024 (n=183).

A total of 183 patients (47.4% of the total sample) were sent an SMS text messaging reminder before their appointment day. Among these SMS text messaging reminder recipients, 143/183 (78.1%) confirmed receipt of the reminders (Table 1). The majority of SMS text messaging reminders (174/183, 95.0%) were sent between 9:00 and 14:00 Monday through Friday (Figure 1).

**Table 1.** Reminder receipt confirmation, Port-au-Prince metropolitan area, 2024.

Means of reminder	Sent	Receipt confirmation, n (%)	
SMS text messaging	183	143 (78.1)	
Email	19	10 (53)	

**Figure 2.**
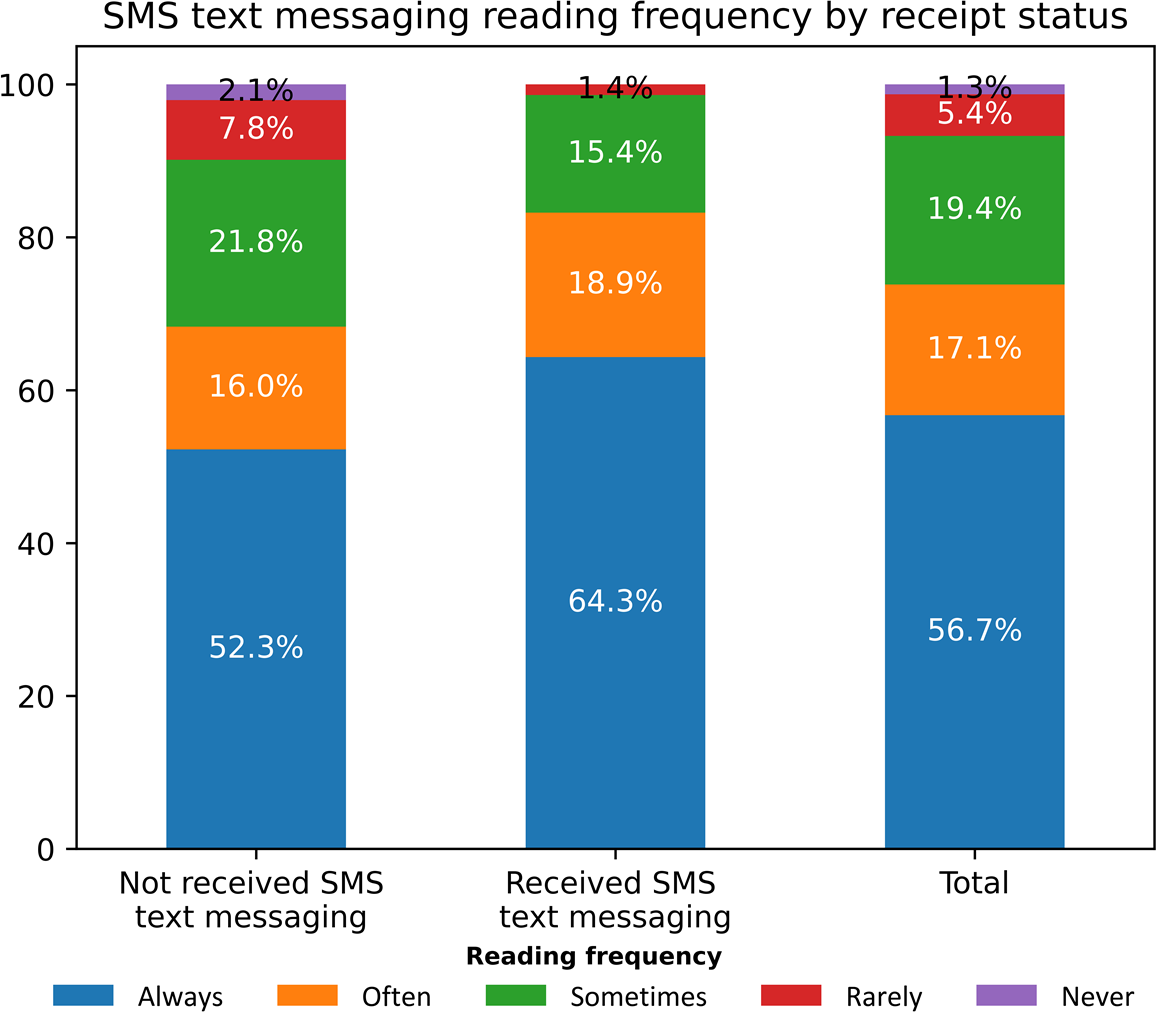
SMS text messaging reading frequency by receipt status, Port-au-Prince metropolitan area, 2024 (n=386).

SMS text messaging reading behavior varied among patients. Overall, 219/386 (56.7%) patients always, 66/386 (17%) often, 75/386 (19%) sometimes, 21/386 (5%) rarely, and 5/386 (1%) never read their SMS text messaging. The proportions of patients who always or often read their SMS text messaging were higher among those who confirmed receipt of reminders compared to those who did not: 92/143 (64%) and 27/143 (19%) versus 127/243 (52.3%) and 39/243 (16%). The trend was reversed for patients who sometimes, rarely, or never read their SMS text messaging. Among patients who confirmed receipt of reminders, 22/143 (15%) sometimes, 2/143 (1%) rarely, and 0/143 (0%) never read their SMS text messaging. And among those who did not confirm receipt of reminders, 53/243 (22%) sometimes, 19/243 (8%) rarely, and 5/243 (2%) never read their SMS text messaging (Figure 2).

### Patient’s Perception Toward Reminders

**Figure 3.**
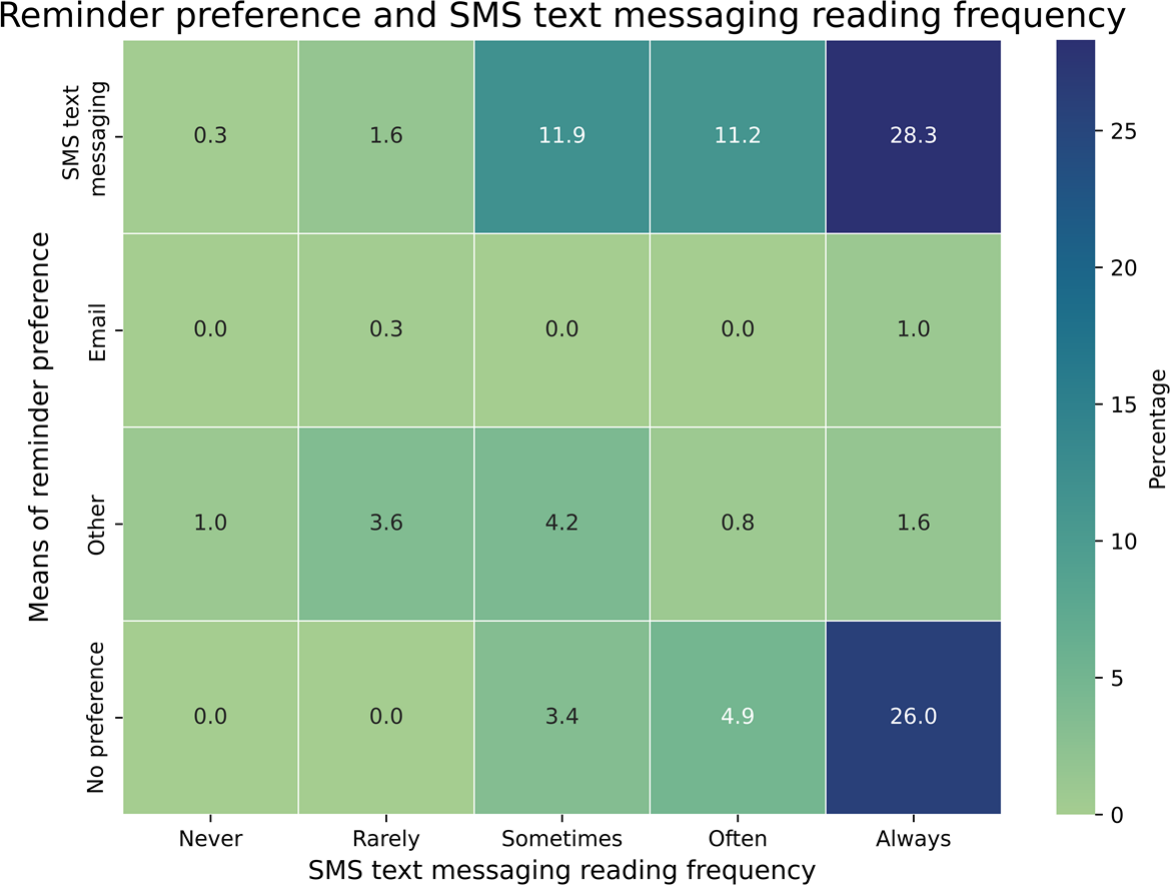
Means of reminder preference and SMS text messaging reading frequency, Port-au-Prince metropolitan area, 2024 (n=386).

All patients (N=386, 100%) liked the initiative of sending reminders before the appointment day and found the reminders helpful. The near-totality of patients (385/386, 99.7%) would like to receive reminders in the future. Of all patients, 271/386 (70%) either had no preference or preferred receiving reminders via SMS text messaging and often or always read their SMS text messaging. In contrast, 19/386 (5%) of patients expressed a preference for reminder methods other than SMS text messaging and rarely or never read their SMS text messaging (Figure 3).

### Late and Nonattendance

**Figure 4.**
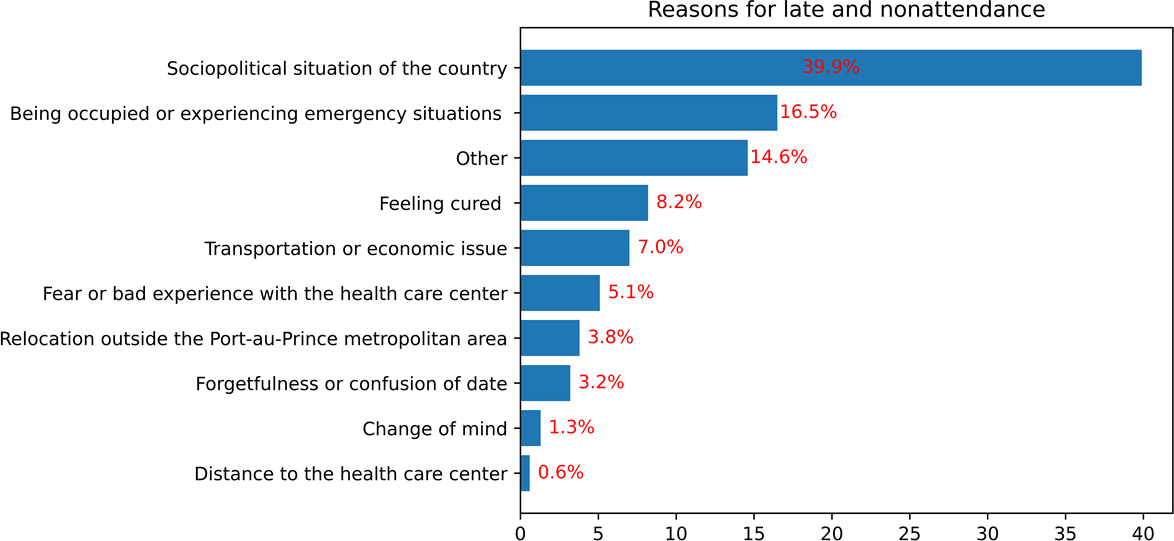
Reasons for late and nonattendance to medical appointment, Port-au-Prince metropolitan area, 2024 (n=158).

Among 158 patients who missed their original appointments, 31/158 (20%) of them voluntarily returned at a later date to see their physician. Most commonly reported reasons for late or nonattendance were the sociopolitical situation of the country (63/158, 40%), being occupied or experiencing emergency situations (26/158, 17%), feeling cured (13/158, 8%), transportation or economic issues (11/158, 7%), fear or bad experiences with the health care center (8/158, 5%), and relocation outside the Port-au-Prince metropolitan area (6/158, 4%) (Figure 4). The majority of patients (26/31, 84%) who voluntarily returned to their appointment did so within 15 days after the original appointment. The remaining 5/31 (16%) returned between 15 and 31 days after the original appointment.

### Overall Attendance Rates and Patient Characteristics

Attendance rates were higher among patients who confirmed receiving a reminder compared to those who did not (114/147 [77.6%] vs 145/239 [60.7%]), with a crude odds ratio (COR) of 2.24 (95% CI 1.40-3.57). Patients with a chronic disease had a lower attendance rate than those without (26/58 [45%] vs 230/324 [71%]), with a COR of 0.33 (95% CI 0.19-0.59). Attendance rates were higher among patients who traveled less than 60 minutes to the health care center 92/129 (71%), 114/152 (75%), and 20/37 (54%) for less than 30 minutes, between 30 and 60 minutes, and 60 minutes or more, respectively. The CORs among these groups were 2.11 (95% CI 1.00-4.48) for less than 30 minutes and 2.55 (95% CI 1.21-5.36) for between 30 and 60 minutes. Attendance rates increased as patient satisfaction with their physicians increased. Attendance rates did not differ significantly among other socioeconomic factors, such as mode of transportation, self-covering of medical expenses, perceived cost of services, occupation, education level, patient age groups, and patients’ sex (Table 2).

**Table 2.** Bivariate analysis of appointment attendance and patient characteristics, Port-au-Prince metropolitan area, 2024.

Patient characteristics	N	n (%)		COR^a^ (95% CI)	
Sample	386	259 (67.1)		—^b^	
Receipt confirmation of reminder					
Yes	147	114 (77.6)		2.24 (1.40-3.57)	
No	239	145 (60.7)		1 (Ref.)	
Time to appointment (in days)					
<8	134	93 (69)		1.24 (0.71-2.17)	
8-15	49	32 (65)		1.03 (0.50-2.12)	
15-22	95	61 (64)		0.98 (0.54-1.78)	
22-30	12	11 (92)		6.03 (0.75-48.74)	
≥30	96	62 (65)		1 (Ref.)	
Being with chronic disease					
Yes	58	26 (45)		0.33 (0.19-0.59)	
No	324	230 (71)		1 (Ref.)	
Travel time (in minutes)					
<30	129	92 (71)		2.11 (1.00-4.48)	
30-60	152	114 (75)		2.55 (1.21-5.36)	
≥60	37	20 (54)		1 (Ref.)	
Unable to estimate	68	33 (49)		0.8 (0.36-1.79)	
Modes of transportation					
Walking	28	18 (64)		0.87 (0.39-1.95)	
Tap-tap	124	82 (66)		0.94 (0.60-1.47)	
Motorcycle taxis	94	59 (62)		0.78 (0.48-1.26)	
Car taxis	19	12 (63)		0.83 (0.32-2.17)	
Private motorcycle	29	20 (69)		1.1 (0.48-2.48)	
Private car	215	149 (69.3)		1.25 (0.82-1.92)	
Self-covering medical expenses					
No	50	32 (64)		1 (Ref.)	
Yes, partially	152	106 (69.7)		1.3 (0.66-2.54)	
Yes, entirely	181	120 (66.3)		1.11 (0.58-2.13)	
Perception of service costs					
Affordable	56	40 (71)		1.36 (0.71-2.61)	
Reasonable	125	87 (70)		1.25 (0.77-2.02)	
Expensive	193	125 (64.8)		1 (Ref.)	
Don’t know	12	7 (58)		0.76 (0.23-2.49)	
Satisfaction with the physician					
≤6	40	19 (48)		1 (Ref.)	
7	66	32 (49)		1.04 (0.47-2.28)	
8	132	96 (73)		2.95 (1.42-6.11)	
9	90	66 (73)		3.04 (1.40-6.61)	
10	58	46 (79)		4.24 (1.74-10.30)	
Occupation of respondents					
Merchant	42	23 (55)		1 (Ref.)	
Self-employed	33	23 (70)		1.9 (0.73-4.96)	
Entrepreneur or Investor	25	17 (68)		1.76 (0.62-4.95)	
Employee	148	109 (73.6)		2.31 (1.14-4.69)	
Managerial staff (employee)	18	12 (67)		1.65 (0.52-5.23)	
Student	44	28 (64)		1.45 (0.61-3.43)	
Other	12	6 (50)		0.83 (0.23-2.98)	
Unemployed	54	33 (62)		1.3 (0.57-2.94)	
Prefer not to answer	10	8 (80)		3.3 (0.63-17.45)	
Education Level of Respondents					
Secondary or less	204	132 (64.7)		1 (Ref.)	
Post secondary	153	108 (70.6)		1.31 (0.83-2.06)	
Prefer not to answer	29	19 (66)		1.04 (0.46-2.35)	
Having children					
Yes	251	175 (69.7)		1.4 (0.90-2.17)	
No	135	84 (62)		1 (Ref.)	
Patient age (years)					
≤16	128	39 (70)		1.16 (0.64-2.10)	
16-30	83	55 (66)		1 (Ref.)	
30-45	121	87 (72)		1.3 (0.71-2.38)	
>45	54	28 (52)		0.55 (0.27-1.11)	
Patient sex					
Female	275	87 (68)		1.22 (0.77-1.94)	
Male	111	71 (64)		1 (Ref.)	

a COR: crude odds ratio.

b Not applicable.

### Socioeconomic Factors Associated With Overall Attendance Rates

**Figure 5.**
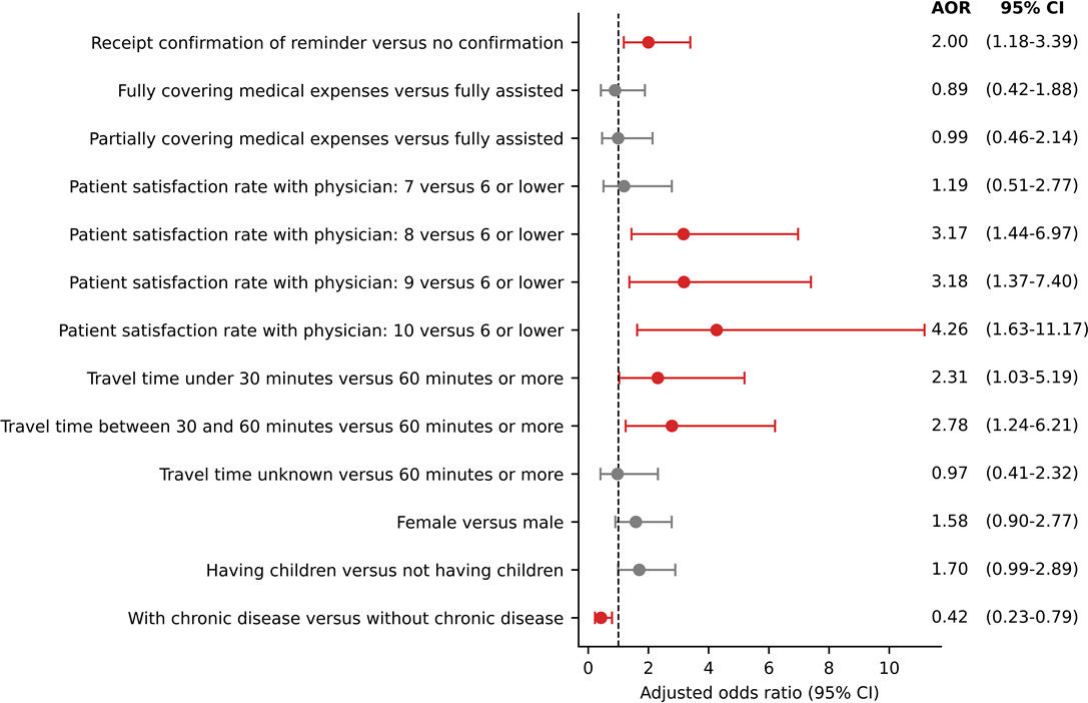
Socioeconomic factors associated with overall attendance rates in Port-au-Prince metropolitan area, 2024. AOR: adjusted odds ratio;

In the multivariate regression analysis, patients who confirmed receiving a reminder were more likely to attend their appointment compared to those who did not (AOR 2.0, 95% CI 1.18-3.39). A patient satisfaction rate of 8 or higher with their physicians was significantly associated with higher attendance rates, compared to 6 or lower, with adjusted odds ratios increasing with satisfaction. Patients traveling less than 60 minutes were more likely to attend their appointment compared to those traveling 60 minutes or more, with AORs of 2.31 (95% CI 1.03‐5.19) for less than 30 minutes and 2.78 (95% CI 1.24‐6.21) for 30-60 minutes. Patients with a chronic disease were less likely to attend their appointment compared to those without (AOR 0.42, 95% CI 0.23-0.79). Fully or partially covering medical expenses, gender, and having children were not associated with attendance rates (Figure 5). The regression result table was provided as well in the appendix ( Multimedia Appendix 2).

## Discussion

### Principal Findings

Through this study, we aimed to assess the effectiveness of text message reminders on patient attendance in the context of the sociopolitical crisis of Haiti. After controlling for socioeconomic factors, we found significantly higher attendance rates among patients who confirmed receiving a reminder compared to those who did not, with an AOR equal to 2.0, suggesting that text message reminders have the potential to improve appointment attendance in Haiti despite the sociopolitical crisis. This finding confirms the results of the previous study, conducted between January 2021 and November 2023, on the effectiveness of reminder systems to improve appointment attendance in the sociopolitical context of Haiti, where the odds of attending appointments were 2.95 for patients who received an SMS text messaging reminder compared to those who did not receive a reminder [16].

### Other Factors Influencing the Effectiveness of Text Message Reminders

The effectiveness of text message reminders has been influenced by a variety of factors, including patient perceptions, behaviors, and socioeconomic conditions. Travel time was independently associated with appointment attendance. Patients traveling less than 60 minutes to their health care centers were more likely to attend their appointments. The burden of travel, both in terms of time and financial cost, can discourage patients from attending appointments, leading to missed opportunities for care and treatment. In addition to being a classic determinant of health care accessibility, travel time as a factor associated with appointment attendance in our study may also be explained by the sociopolitical crisis. In a sociopolitical crisis where armed gangs control the majority of the Port-au-Prince metropolitan area, every minute spent outside increases the risk of being the victim of a kidnapping or terrorist attack [1]. A study following up on nonattendance at a teaching hospital in a semiurban city in southwestern Nigeria in 2023 reported that distance to the hospital was an independent predictor of nonattendance [21].

Perception of physician service quality was a key determinant of patient appointment attendance. Patient satisfaction with their physician was significantly associated with higher attendance rates, with AOR increasing with satisfaction. In addition, 5% of patients who missed their original appointment reported that they did not return because of fear or a bad experience with the health care center. Patient satisfaction has previously been reported to be a significant determinant of attendance at medical appointments. A study on the relationship between patient experience and quality and use of primary care services, including 8355 primary care patients from 22 sites in the United States between January 2012 and July 2014, found a significant association between patients’ reported experiences with their providers and their likelihood of attending scheduled appointments. Positive interactions with providers were associated with lower no-show rates, highlighting the impact of patient satisfaction on attendance [22]. A meta-analysis on physician communication and patient adherence to treatment, including 127 studies in 2009, reported a 19% higher risk of nonadherence among patients whose physician communicates poorly than among patients whose physician communicates well [23].

Patients with a chronic disease were less likely to attend their appointments. Patients with chronic conditions often face challenges that can impact their attendance at medical appointments [24,25]. However, a study on failure to keep appointments at a community health center in Israel reported a lower nonattendance rate among patients with a chronic disease requiring follow-up compared to patients with an acute disease (8% vs 43% for children, 21% vs 38% for adults, and 28% versus 83% for older adults, respectively) [26]. In contrast, in situations of sociopolitical crisis, where going outside is highly life-threatening, the trend is reversed, suggesting that those who attended their appointments in these circumstances had a more acute illness and required emergency care.

### Attendance Trends and Voluntary Return

The overall attendance rate improved significantly over time, increasing to 67.1%, which is 4 times higher than the 17.6% rate observed in a previous study on the effectiveness of a patient reminder system in the Port-au-Prince metropolitan area, conducted between January 2021 and November 2023 [16]. This improvement can be partially explained by a higher implementation of the reminder system. The proportion of SMS text messaging reminders sent to patients increased from 8.1% to 47.4% [16]. The other reason that may explain this finding is the fact that the earlier study was based only on attendance at the appointment day, while the later study took into account patients who voluntarily returned to their appointment at a later date after missing the original one.

We found that 20% of patients who missed their original appointments voluntarily returned at a later date. This finding is substantially lower than that reported in a study on the reasons for missed appointments among patients with stable chronic conditions who needed medication refills, conducted in South Africa between 2014 and 2015 [25]. The authors reported that 67% of patients who missed their original appointments voluntarily returned at a later date to obtain medicines. This difference may be due to the sociopolitical crisis in Haiti. Because of terrorist attacks and the high risk of kidnapping, people are afraid to venture into the streets for their activities. In our study, the sociopolitical crisis in the country was reported by 40% of patients who missed their original appointment as a reason for late or nonattendance. Displacement as a result of the sociopolitical crisis may also explain this difference. It was reported that approximately one in ten of the population was displaced due to gang violence [1]. Additionally, 4% of patients who missed their original appointment attributed their absence to relocation outside the Port-au-Prince metropolitan area.

### Acceptability of Text Message Reminders

The patient population in the Port-au-Prince metropolitan area is very receptive to appointment reminders, despite the sociopolitical crisis of Haiti. All patients liked the initiative of sending reminders before the appointment day and found the reminders helpful. The near-totality of patients would like to receive reminders in the future. A pilot study assessing the feasibility and acceptability of text message reminders among patients with chronic noncommunicable diseases in a rural health care facility in Haiti found that all reminder recipients liked the idea of receiving reminders, and 92.7% expressed a desire to receive further messages [27]. SMS text messaging appears to be a promising tool for increasing the reach of text message reminders in Haiti. We found that more than two-thirds of patients enrolled in the study often or always read their text messages, and only 1% of patients never read their SMS text messaging. This finding is substantially higher than that reported by other studies in low- and middle-income countries. A study to understand the sociodemographic factors associated with intention to receive SMS text messages for health information in Bangladesh reported that less than half of the patients who owned a mobile phone could read SMS text messaging [28]. In Kenya, a national survey on the ownership and use of mobile phones among health workers and patients reported that 71.4% of patients who owned a phone used SMS text messaging [10].

Despite the fact that most SMS text messaging reminders were sent on weekdays during busy hours, 78.1% of SMS text messaging reminder recipients confirmed receipt of the reminders. SMS text messaging reminders appear to be more widely accepted in Haiti than in some other settings. A randomized controlled trial designed to assess the effectiveness, feasibility, and acceptability of SMS text messaging and consultation to manage the blood pressure of Chinese patients with hypertension reported that only 68% of the patients reported reading the messages sent by the researchers. And 60% of those who did not read the messages reported having not read them, as they did not have the habit of reading messages [29]. However, unlike our study, the patient population in the previously cited study consisted only of patients with hypertension.

### Limitations and Recommendations

Our study had few limitations. The retrospective nature of the study may introduce a potential recall bias. However, patients were interviewed within a 6-month window around their appointments. This balanced timeframe allowed time for them to return at a later date if they had missed the original appointment and was recent enough to minimize the risk of forgetting information related to the appointment. Our study was conducted in 2 private health care centers. Given the sociopolitical crisis, which is a key aspect of the study context, affects individuals regardless of socioeconomic status, we believe our sample is representative of the population in the Port-au-Prince metropolitan area [1,30]. The SMS text messaging system was not consistently deployed throughout the study, as the health care centers occasionally failed to recharge their SMS text messaging accounts on time. However, the system did not discriminate in who reminders would be sent to: all patients were sent a reminder when the system was recharged, and no SMS text messaging reminders were sent when the system was out of credit. This provided a reliable comparison group and strengthened the robustness of our findings. While the reasons for patient appointments were unknown, which could have influenced whether or not a patient attended their appointment, the nondiscriminatory nature of the reminder system meant that this potential bias was probably nondifferential between those who received reminders and those who did not. Of the respondents, 37% did not agree to participate in our study. It is possible that these are mainly those who did not attend their appointment. However, our sensitivity analysis showed that this potential nonresponse bias is likely to be nondifferential between those who confirmed receipt of reminders and those who did not, and therefore does not alter the measure of association.

To enhance appointment adherence in Haiti or crisis-affected settings, health care centers should consider integrating text message reminder systems into their patient management workflows, ensuring that messages are personalized and delivered in a timely manner. Additionally, efforts should be made to address barriers such as travel time and patient experience with the health care system, as these factors significantly influence attendance and treatment adherence.

### Conclusion

Appointment attendance was influenced by receipt status of text message reminder, travel time, patient satisfaction with their physician, and chronic conditions in the Port-au-Prince metropolitan area. The overall acceptability and effectiveness of SMS text messaging suggest that they can be a valuable tool in health care settings to improve appointment attendance despite sociopolitical crises, especially when adapted to the local context and individual needs. We recommend that health care centers in Haiti consider integrating SMS text messaging reminder systems into routine patient management to enhance adherence and optimize care delivery.

## Supplementary material

10.2196/77010 Multimedia Appendix 1 Nonresponse analysis, using appointment data from the reminder system.

10.2196/77010 Multimedia Appendix 2 Table regression from the multivariate logistic model. It is a supplement to the forest plot provided in the main manuscript.

10.2196/77010 Checklist 1 Strengthening the Reporting of Observational Studies in Epidemiology (STROBE) checklist

## Abbreviations

AOR: adjusted odds ratio
COR: crude odds ratio
STROBE: Strengthening the Reporting of Observational Studies in Epidemiology
