# Supplementary material for: Acceptability and Effectiveness of Text Message Reminders to Improve Patient Attendance During the Sociopolitical Crisis in Haiti: Telephone-Based Survey
Source: JMIR Form Res. 2026 Feb 23;10:e77010. doi: 10.2196/77010 (PMC12928543; doi:10.2196/77010)
Supplement: Multimedia Appendix 1 [file formative-v10-e77010-s001.pdf]

Non-response analysis, using appointment data from the reminder system

Of the 613 patients contacted, 227 did not complete the survey, resulting in a non-response rate of 37.0%. The non-response rate did not differ significantly between the reminder groups, being 37.7% for patients to whom a reminder and 36.8% for patients to whom no reminder was sent (p-value=0.95). The non-response rate did not differ by clinical specialty (p-value 0.89), gender (p-value 0.26), or age group (p-value 0.67) (Table S1).

Table S1. Comparaison of non-response rates between socio-demographic groups

	N	n	%	p-value
Sample	613	227	37.0	-
SMS reminder recipient	-	-	-	0.95
Yes	292	109	37.3	-
No	321	118	36.8	-
Clinical specialty	-	-	-	0.89
Gynecology-Obstetrics	253	95	37.5	-
Internal Medicine	143	53	37.1	-
Orthopedics	16	5	31.3	-
Pediatrics	184	66	35.9	-
Other	17	8	47.1	-
Gender	-	-	-	0.26
Female	447	172	38.5	-
Male	166	55	37.0	-
Age group	-	-	-	0.67
<=16	194	66	34.0	-
]16, 30]	134	51	38.1	-
]30, 45]	193	72	37.3	-
>45	92	38	41.3	-

Non-response rates were not statistically different between reminder and sociodemographic groups. Although non-respondents may be primarily those who did not attend their appointment, the difference in attendance rates between those who confirmed receipt of reminders and those who did not is likely to be nondifferential.

Additional file 1. Non-response analysis, using appointment data from the reminder system
