# Supplementary material for: Acceptability and Effectiveness of Text Message Reminders to Improve Patient Attendance During the Sociopolitical Crisis in Haiti: Telephone-Based Survey
Source: JMIR Form Res. 2026 Feb 23;10:e77010. doi: 10.2196/77010 (PMC12928543; doi:10.2196/77010)
Supplement: Multimedia Appendix 2 [file formative-v10-e77010-s002.pdf]

Table regression from the multivariate logistic model. It is in supplement to the forest plot provided in the main manuscript.

Table S2. Socio-Economic Factors Associated with Overall Attendance Rates

	N	n	%	$OR_{(crude)}$	(95% CI)[a]	$OR_{(adjusted)}$	(95% CI)[b]
Sample	386	259	67.1	-	-	-	-
Receipt Confirmation of Reminder							
Yes	147	114	77.6	2.24	(1.40, 3.57)	2.0	(1.18, 3.39)
No	239	145	60.7	1 (Ref.)	-	1 (Ref.)	-
Being with Chronic disease							
Yes	58	26	45	0.33	(0.19, 0.59)	0.42	(0.23, 0.79)
No	324	230	71	1 (Ref.)	-	1 (Ref.)	-
Travel time (in minutes)							
<30	129	92	71	2.11	(1.00, 4.48)	2.31	(1.03, 5.19)
[30 - 60[	152	114	75	2.55	(1.21, 5.36)	2.78	(1.24, 6.21)
>=60	37	20	54	1 (Ref.)	-	1 (Ref.)	-
Unable to estimate	68	33	49	0.8	(0.36, 1.79)	0.97	(0.41, 2.32)
Self-covering medical expenses							
No	50	32	64	1 (Ref.)	-	1 (Ref.)	-
Yes, partially	152	106	69.7	1.3	(0.66, 2.54)	0.89	(0.42, 1.88)
Yes, entirely	181	120	66.3	1.11	(0.58, 2.13)	0.99	(0.46, 2.14)
Satisfaction with the physician							
<=6	40	19	48	1 (Ref.)	-	1 (Ref.)	-
7	66	32	49	1.04	(0.47, 2.28)	1.19	(0.51, 2.77)
8	132	96	73	2.95	(1.42, 6.11)	3.17	(1.44, 6.97)
9	90	66	73	3.04	(1.40, 6.61)	3.18	(1.37, 7.40)
10	58	46	79	4.24	(1.74, 10.30)	4.26	(1.63, 11.17)
Patient Gender							
Female	275	87	68	1.22	(0.77, 1.94)	1.58	(0.90, 2.77)
Male	111	71	64	1 (Ref.)	-	1 (Ref.)	-

n: number of patients who attended their appointments;
$OR_{(crude)}$: *crude Odds Ratio;*
a: Confidence Interval for crude Odds Ratios
$OR_{(adjusted)}$: *adjusted Odds Ratio;*
b: Confidence Interval for adjusted Odds Ratios

Additional file 2. Table regression of the multivariate logistic regression model
